# A Case Series: Continuous Kidney Replacement Therapy in Neonates With Low Body Weight

**DOI:** 10.3389/fped.2021.769220

**Published:** 2021-11-17

**Authors:** Chen-Yu Wu, Yung-Chieh Lin, Chih-Chia Chen

**Affiliations:** ^1^Department of Pediatrics, National Cheng Kung University Hospital, College of Medicine, National Cheng Kung University, Tainan, Taiwan; ^2^Department of Pediatrics, College of Medicine, National Cheng-Kung University, Tainan, Taiwan; ^3^Graduate Institute of Clinical Medicine, College of Medicine, National Cheng-Kung University, Tainan, Taiwan

**Keywords:** acute kidney injury, dialysis, low body weight, continuous kidney replacement therapy (CKRT), neonate

## Abstract

Emerging data indicate that acute kidney injury (AKI) may contribute to a worse prognosis in the infant population. Kidney replacement therapy (KRT) can be used to treat patients with AKI; however, this technique is challenging in patients in the neonatal intensive care units (NICUs) due to the low body weights and blood volumes in this population. Peritoneal dialysis (PD) is a potential modality since it is technically less challenging. However, PD has been associated with several disadvantages, including poor fluid status control, catheter-associated leakage, and peritonitis. Unfortunately, these complications can cause the temporary cessation of PD. Continuous kidney replacement therapy (CKRT) may represent a suitable alternative for PD. CKRT may be technically feasible in infants; however, little is known about the application of CKRT in neonates with low body weights. In this report, we discuss three cases of CKRT who were treated in the NICU at a tertiary medical center in southern Taiwan. We selected an adequate catheter diameter and achieved vascular access *via* an internal jugular vein or umbilical vein. The prescription of an appropriate dose of heparin was then used to prolong the circuit life of the CKRT. The maintenance of circuit durability in neonates with low body weight remains problematic. We hope that our experience can assist with the future clinical management of CKRT in neonates with low body weight.

## Introduction

Approximately 30% of critically ill neonates experience acute kidney injury (AKI) (1), a condition that is associated with a high rate of mortality and morbidity (2). Some of these patients may require kidney replacement therapy (KRT) to deal with complications. Acute peritoneal dialysis (PD) is a common form of management for such patients due to ease of accessing (3, 4). However, PD is not able to effectively manage cases involving severe fluid overload (5). In addition, PD has also been associated with a number of complications, including catheter-related leakage at the insertion sites and infection of the peritoneal cavity (6). During infection, it is necessary to terminate PD on a temporary basis while applying hemodialysis. Furthermore, patients who do not recover from AKI may need some form of bridging therapy during the break-in periods after the permanent implantation of a PD catheter (7). Traditional hemodialysis might result in unstable hemodynamics in the neonatal population and is not appropriate for this population. Continuous KRT (CKRT) may represent a suitable alternative. However, very few studies have investigated the application of CKRT in neonates with low body weights (<2.5 kg) (8–13) (Supplementary Table 1). In this report, we discuss our experiences of three neonates with a low birth weight who were treated with CKRT at a tertiary medical center in southern Taiwan.

## Clinical Cases

A summary of all three cases is provided in Tables 1, 2.

**Table 1:** Clinical characteristics of our three cases.

	**Case A**	**Case B**	**Case C**
Gender	Male	Female	Female
Gestational age	35 weeks	33 weeks	38 weeks
Birth body weight	1,675 g	1,500 g	1,800 g
Body weights when CKRT	1,892 g	1,835 g	1,960 g
Etiologies for renal insufficiency	Acute tubular necrosis	CAKUT	Acute tubular necrosis
Extrarenal anomaly		Right disc coloboma, anterior ectopic anus, absence of vagina and uterus	Type II ventricular septal defect (0.65 cm) and severe coarctation of aorta

*CAKUT, congenital anomalies of the kidney and urinary tract; CKRT, continuous kidney replacement therapy*.

**Table 2:** Continuous kidney replacement therapy setting of our three cases.

	**Case A**		**Case B**	**Case C**	
	**Course 1**	**Course 2**		**Course 1**	**Course 2**
Vascular access site	Umbilical vein	Umbilical vein	Right IJV	Umbilical vein	*A site*: right IJV
					*V site*: right femoral vein
Catheter	7 Fr. dual lumen CVC	8 Fr. Hemocath	5.5 Fr. three lumen CVC	8 Fr. Hemocath	two 5.5 Fr. CVC
Machines			HF 400 (Infomed)
Blood flow	6.6 ml/kg/min	10 ml/kg/min	5–6.5 ml/kg/min	10 ml/kg/min	5–6 ml/kg/min
Ultrafiltration	26 ml/kg/h	34 ml/kg/h	50 ml/kg/h	34 ml/kg/h	38–57 ml/kg/h
Predilution ratio	50%	50%	50%	50%	50%
Anticoagulation^a^	–	–	Heparin	–	Heparin
			*Loading*: 14–15 IU/kg		*Loading*: 10 IU/kg
			*Maintain*: 8–9 IU/kg/h		*Maintain*: 5 IU/kg/h
Sessions	7	5	37	10	29
Mean circuit survival, hours, mean [range]	11.5 [0.5–49.5]	23.6 [8.1–71.9]	11.7 [0.3–52.3]	11.8 [0.4–26.8]	32.1 [1.9–72.1]

*CVC, central venous catheter; IJV, internal jugular vein.*

a *Heparin was used to keep activated clotting time within 150–200 s*.

### Case A

A 2-day-old male neonate was referred to our neonatal intensive care unit (NICU) due to anuria for more than 48 h. He was born by cesarean section at 35 weeks of gestation at a regional hospital and weighed 1,675 g at birth. Antenatally, his mother had developed oligohydramnios; this was related to the premature rupture of membranes during the second trimester. At birth, the patient suffered from meconium aspiration, cyanosis, and bradycardia. Following resuscitation, his heart rate improved; Apgar scores were 5 and 7 at 1 and 5 min, respectively. A septic workup was performed, and the patient was administered antibiotics (vancomycin and ceftazidime) prior to referral based on their antibiotic policy. The patient was then referred to our hospital for further management.

On arrival, his vital signs were stable. However, fluid overload was noted (body weight: 1,892 g; 13% fluid overload). General edema was noted without skin rash. Laboratory analysis showed no evidence of leukocytosis (13,400/cumm), left shifting (Band: 1%, Seg: 77%), or eosinophilia (1%). Biochemical evaluations revealed elevated levels of creatinine (4.81 mg/dl) and blood urea nitrogen (28 mg/dl), hyponatremia (114 mmol/L), and non-anion gap metabolic acidosis (HCO_3_: 17.2 mmol/L). Renal ultrasound revealed increased renal echogenicity bilaterally without structural abnormality of the kidneys and bladder. We suspected acute tubular necrosis.

Intravenous furosemide was provided for diuresis, although the response was poor. Due to the overload of refractory fluid, we decided to commence continuous venovenous hemofiltration (CVVH) therapy. One 7-Fr dual lumen central venous catheter (CVC) (ARROW^®^ Teleflex, Wayne, PA, USA) was placed in his umbilical vein under ultrasound guidance to provide vascular access; radiography was used to confirm the position of the catheter's tip. Heparin solution (10 U/ml) was locked in CVC before initiation of CKRT. Before the catheter was connected to the machine, the dialysis circuit was rinsed with heparin and primed with packed red blood cells (hematocrit: 70–80%) to combat low body weight. No more heparin was given because his activated clotting time (ACT) was 180–230 s initially. Blood flow was initially set to 12 ml/min (6.6 ml/kg/min). The filtration fraction was 0.1–0.2, and ultrafiltration was initially 50 ml/h (26 ml/kg/h). In total, 50% of the replacement fluid was given as predilution; the remainder was given as postdilution. The net ultrafiltration rate was set based on the infusion fluid to remove fluid gently. Due to the patient's coagulopathy, no anticoagulant was given initially.

**Figure 1:**
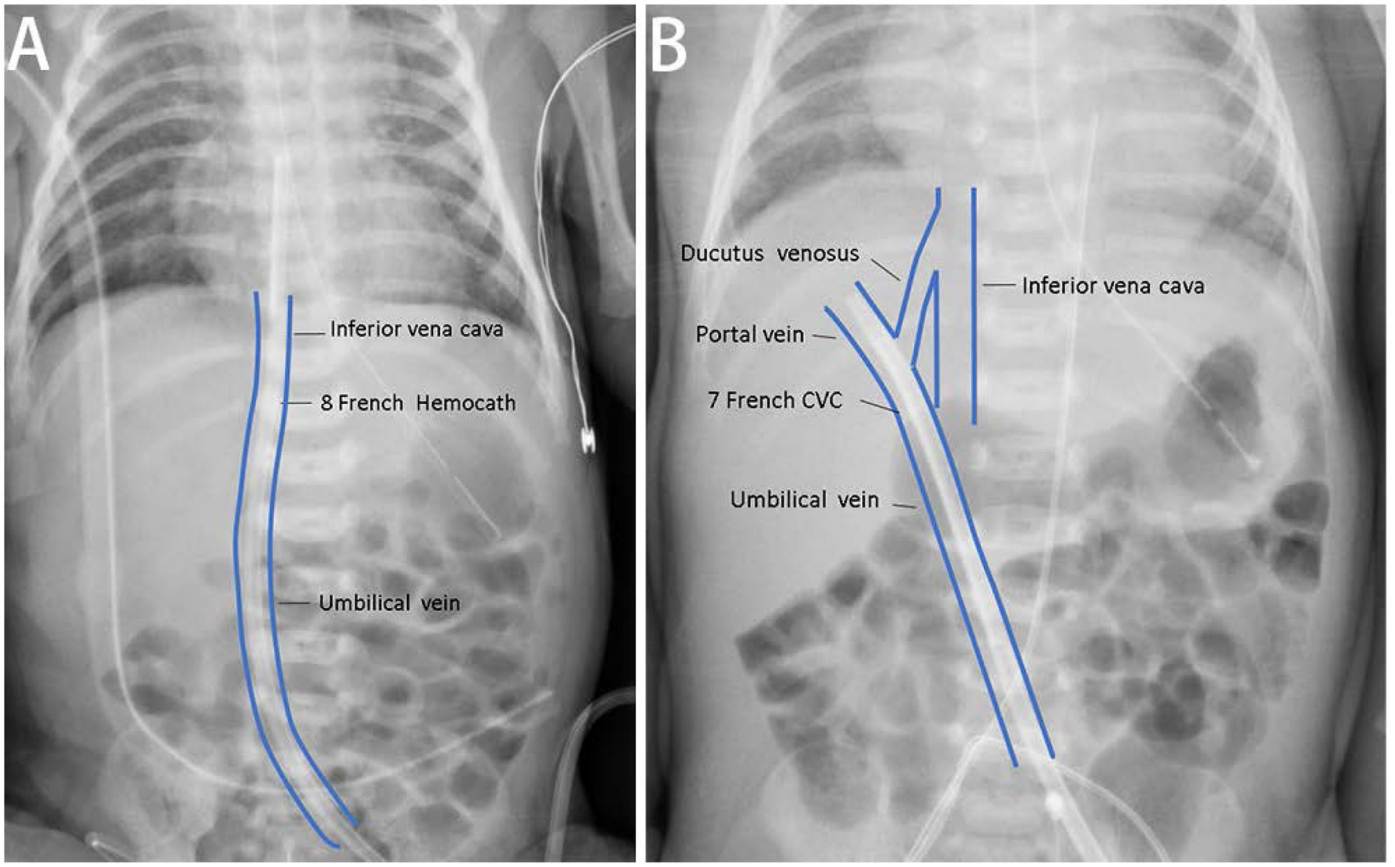
The correct and incorrect positions of vascular accesses of continuous kidney replacement therapy (CKRT). **(A)** For case A, we placed one 8-Fr Hemocath^®^ *via* the umbilical vein, and the location of the catheter tip was appropriated. **(B)** We also tried to establish vascular access *via* the umbilical vein for case B. The catheter tip was at the portal vein and could not provide satisfactory duration for dialysis.

Initially, the 7-Fr CVC provided an appropriate circuit life span of 49.5 h but varied from 0.5 to 12 h in subsequent courses. Therefore, when guiding the wire, we changed the vascular access to a larger 8-Fr Hemocath (MedCOMP^®^ Medical Components, Inc., Harleysville, PA, USA) that was placed in the umbilical vein (Figure 1A). A permanent PD tube was also inserted preemptively by a pediatric surgeon since it was not possible to predict the recovery of renal function. CKRT was continued as a bridging therapy. We increased the blood flow to 20 ml/min (10 ml/kg/min) thereafter, and the circuit life span ranged from 12 to 71.9 h.

Urinary output and laboratory data improved after CVVH. Therefore, we stopped dialysis. The infant was subsequently discharged, and the PD tube was removed.

### Case B

A female twin B was referred to our institute due to fluid overload for 7 days since birth. She was born by cesarean section at 33 weeks of gestation at a regional hospital and weighed 1,500 g at birth. Her mother had severe preeclampsia and oligohydramnios. Apgar scores were 5 and 7 at 1 and 5 min, respectively. Due to respiratory distress after birth, we applied continuous positive airway pressure. A septic workup was performed, and intravenous antibiotics (ampicillin/sulbactam and gentamicin) were administered. Although the urinary output was fair (2–4 ml/kg/h), we also noted fluid overload (22%). Initial biochemical analysis revealed that serum creatinine was 3.15 mg/dl, blood urea nitrogen was 46 mg/dl, and serum sodium was 115 mmol/L.

Renal ultrasound showed hydronephrosis with a hydroureter on the right side and an ectopic hypodysplastic left kidney in the lower abdomen showing parenchymal changes. Magnetic resonance urography revealed similar findings. Next, we attempted percutaneous nephrostomy (PCN) to release hydronephrosis on the right side. However, very little urine emerged from the PCN, thus suggesting poor renal function on the right. Consequently, we removed the PCN.

Due to progressively deteriorating renal function, KRT was indicated. CKRT was initially selected to avoid dialysate leakage from the previous PCN wound. We tried to establish vascular access *via* the umbilical vein with a 7-Fr CVC (ARROW^®^) catheter; however, this failed was due to the tip of the catheter being located in the portal vein (Figure 1B). Therefore, we attempted PD. However, 2 days later, PD peritonitis occurred along with ultrafiltration failure. On day 11 after birth, a 5.5-Fr three lumen CVC (ARROW^®^) catheter was placed in her right internal jugular vein. Heparin solution (10 U/ml) was locked in CVC before initiation of CKRT. We set the end hole of the CVC as the A site and the distal side hole of the CVC as the V site for CVVH access. The blood flow was 10–12 ml/min (5–6.5 ml/kg/min), and the ultrafiltration rate was 90 ml/h (50 ml/kg/h). Heparin was administered at a loading dose of 14–15 IU/kg and a maintenance dose of 8–9 IU/kg to maintain an ACT of 150–200 s. In total, 50% of the replacement fluid was given as predilution, while the other 50% was given as postdilution. We set the net ultrafiltration rate to remove fluid gradually. Due to frequent hypotension at the beginning of each dialysis session, we gave the patient fluid with 0.9% saline or 5% albumin prior to each session (10 ml/kg). The mean circuit life span was 11.7 h (range: 0.3–52.3 h). Once the peritonitis had recovered, we recommenced PD (from around 1 month after birth).

### Case C

A 2-day-old girl was referred to our institute due to congenital heart disease. She was born at full term with a birth weight of 1,800 g. Two days after birth, we noted a grade II heart murmur in the left paravertebral interscapular area. An echocardiogram revealed patent ductus arteriosus, a type II ventricular septal defect (VSD), and severe coarctation of the aorta. The blood pressures at the upper and lower extremities were initially normal, as was urinary output.

On arrival, biochemical analyses revealed elevated levels of creatinine (1.46 mg/dl) and blood urea nitrogen (19 mg/dl). Computed tomography angiography revealed an almost interrupted coarctation of the aorta. We administered prostaglandin E1 to retain patency in the ductus arteriosus. She received total correction of coarctation and VSD on day 4 after birth. Subsequently, the patient experienced hypotension (mean artery blood pressure: 37 mmHg); this was treated with inotropic agents. The patient also experienced refractory anuria. We decided to perform early KRT and selected CKRT due to the better likelihood of achieving a net negative fluid balance. One 8-Fr Hemocath (MedCOMP^®^) catheter was placed in her umbilical vein to provide vascular access. The initial CVVH settings involved a blood flow of 20 ml/min (10 ml/kg/min) and an ultrafiltration rate of 65 ml/h (34 ml/kg/h). In total, 50% the replacement fluid was given prior to filter. Initially, we did not administer anticoagulants. The mean circuit life span was 11.8 h (range: 0.4–26.8 h). However, the vascular access became obstructed 2 days later because of umbilicus infection. Next, we inserted 5.5-Fr CVC three lumen (ARROW^®^) catheters in her right internal jugular vein and right femoral vein. Heparin solution (10 U/ml) was locked in CVC before initiation of CKRT. Next, we used the tip hole of the catheter to aspirate and return blood to her body. We reduced the blood flow to 5–6 ml/kg/min for a small catheter diameter and increased the ultrafiltration rate to 100–150 ml/h (38–57 ml/kg/h) for better clearance. Heparin was administered at a loading dose of 10 IU/kg and a maintenance dose of 5 IU/kg to maintain an ACT of 150–200 s. The mean circuit life span was 32.1 h (range: 1.9–72.1 h). The net ultrafiltration rate was set so that we could mildly remove fluid. The renal function data improved. However, she suffered from severe bloodstream sepsis on day 36 of CVVH therapy and died.

## Discussion

Our experience indicates that CKRT can be conducted in neonates with low body weight. It is well-known that CKRT is associated with several difficulties in neonates. Herein, we provide our positive experiences of this technique. Our experiences may help to guide AKI management in this delicate population of patients in non-specialized hospitals with little experience in this field.

### Comparisons Between Peritoneal Dialysis and Continuous Kidney Replacement Therapy

**Figure 2:**
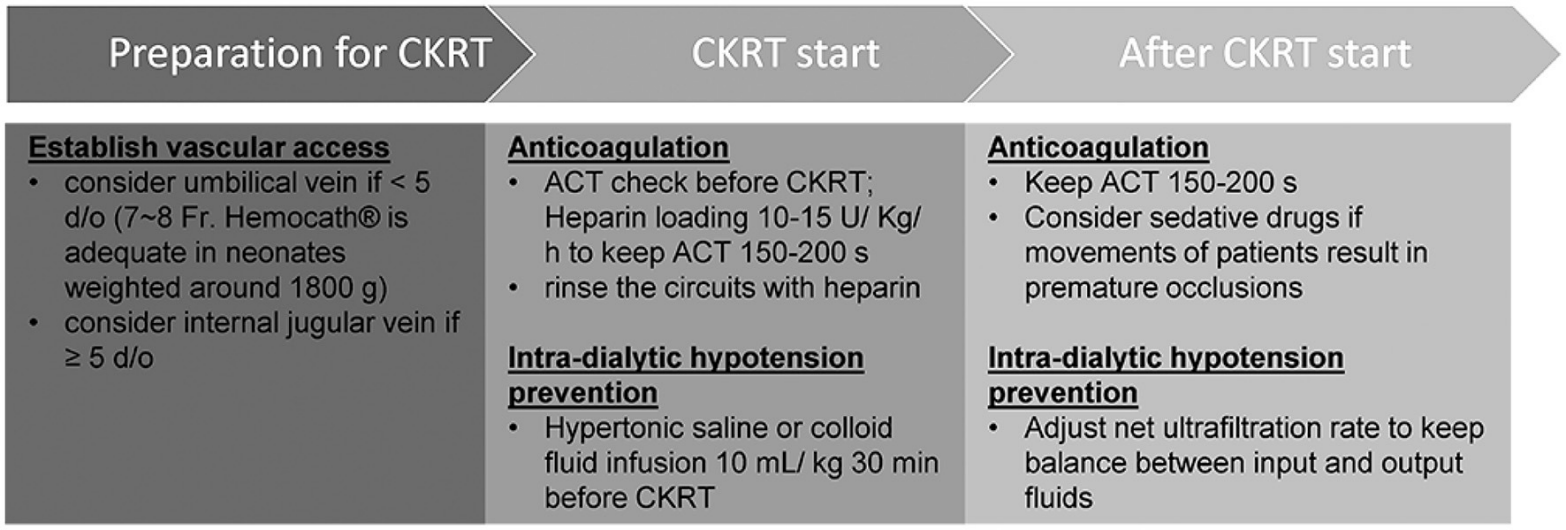
Modified protocol of continuous kidney replacement therapy for neonates with low body weights.

PD is the preferred treatment modality with which to manage AKI in neonates with low body weight (14); however, there are some circumstances where this technique may not be appropriate. For example, Case B received PCN prior to dialysis; thus, fluid leakage was a concern. Peritonitis and ultrafiltration failure may also impede PD [6], as witnessed in Case B. Following the permanent implantation of a PD catheter in Case A, we needed to wait for 14 days to allow the surgical wound to heal before PD started to prevent the leakage of fluid. In addition, PD is unable to manage severe fluid overload in an effective manner, especially when peritoneal perfusion is poor (5, 6). CKRT is not only an alternative modality to manage these situations, but it also represents a bridge that can be deployed when PD sessions are suspended. However, there are many challenges in the application of CKRT on neonates with low body weights, including adequate circuit life span and the prevention of intradialytic hypotension. Although some previous reports have described the use of CKRT in infants with variable body weights, these earlier studies did not focus on the specific management and clinical outcomes of neonates with low body weight (Supplementary Table 1) (8–13). In this report, we describe three neonates with low body weights that were successfully treated with CKRT using our modified protocols (Figure 2).

### Equipment Requirements for the Use of Continuous Kidney Replacement Therapy in Neonates With Low Birth Weights

To improve circuit survival time, we were very careful to select an appropriate size of catheter. We used 8-Fr Hemocath catheters for neonates weighing 1,675–1,800 g. Previous studies reported that large catheter diameters were of benefit to circuit survival (15). In contrast, other researchers have reported that a larger catheter may reduce circuit survival (16). Our experience indicated that 8-Fr Hemocath catheters can be used successfully in neonates with similar birth weights when applied *via* the umbilical vein.

The establishment of vascular access for CKRT in neonates with low body weight can be very challenging. In neonates, the umbilical vein is a strong candidate for vascular access. We successfully established vascular access *via* the umbilical vein in two of our patients but failed to establish access in the third patient due to catheter occlusion and a suspicious infection. Previous reports have highlighted that vascular access *via* the umbilical vein is only suitable in newborns for the first 4–5 days (17). We established vascular access in Case C by using the internal jugular vein as the arterial site and the femoral vein as the venous site; this strategy increased the circuit life of CKRT (mean circuit life: 32.1 h). This result was compatible to a previous study that stated that vascular access *via* the internal jugular vein led to a longer circuit survival time than other techniques (16).

### Anticoagulation During Continuous Kidney Replacement Therapy in Neonates With Low Birth Weights

Anticoagulation played an important role in preventing premature circuit occlusions. Data emerging from adult patients show that citrate is safe and effective for anticoagulation (18, 19); however, little is known about anticoagulation in neonates. Heparin is widely used for anticoagulation in the neonatal population (20). For example, Castillo et al. demonstrated that a higher dosage of heparin maintained an ACT of 150–200 s and that this was associated with a longer circuit life span (21). We applied heparin for anticoagulation in Case B and Case C to maintain the ACT to 150–200 s. We did not administer heparin to Case A because his ACT was 180–230 s initially. In addition, we modified our protocols to further prevent coagulations. First, we rinsed the circuits with heparin without a saline wash. Second, we prescribed sedative drugs during the CKRT session because we noted that patient movement might result in premature occlusions. Finally, we avoided blood transfusions during CKRT sessions (22).

### Methods to Prevent Intra-Dialytic Hypotension in Neonates With Low Birth Weights

Hypotension is the most common complication during CKRT in neonates (23); this is due to the significant extracorporeal volume compared with blood volume. Previous researchers have developed techniques to deal with intra-dialytic hypotension, including lower ultrafiltration rates or blood flow agents (12, 24). However, these methods may reduce clearance effects. We used colloid fluid to prime the circuit, as suggested previously (25). In addition, we used 0.9% saline or 5% albumin for 30 min prior to the CKRT session; we found that blood pressure remained stable when using this protocol, as described in the adult group (26). It is important to give fluids to patients experiencing fluid overload with caution because fluid overload is associated with a poor prognosis (27). We only provided a low volume of fluid (10 ml/kg) to prevent intra-dialytic hypotension, an independent risk factor for mortality (28). Interestingly, novel items of equipment, such as the Cardio-Renal Pediatric Dialysis Emergency Machine (CARPEDIEM) (Italy) and the Newcastle infant dialysis and ultrafiltration system (Nidus) (UK), have been designed for neonates and children (29, 30). These two machines have a lower circuit volume and could help to reduce hypotension in neonates (Supplementary Table 2). We look forward to their popularization over the coming years.

The target range for net ultrafiltration rate still remains controversial. One previous study demonstrated that a higher net ultrafiltration rate was associated with a higher mortality rate (31). However, another study revealed an association between higher net ultrafiltration and improved survival rate (32). Similarly, the association between net ultrafiltration rate and renal recovery rate has yet to be established (33). Other factors, such as fluid overload status and input fluid volumes, may influence clinical outcomes and should also be carefully considered (34). We carefully regulated the net ultrafiltration rate to maintain fluid balance in all three of our cases, rather than focusing on the net ultrafiltration rate only.

### Limitations

Although we provide valuable technical information, there are some limitations to our study. First, we only enrolled three patients; their body weights during CKRT were approximately 1.8 kg. We cannot confirm that these principles are suitable for application in neonates with even lower body weights. In addition, because of the low incidence of neonates with low body weight requiring dialysis, we were only able to report three cases. Further studies, featuring well-designed trials and data from multiple centers will be required in the future.

## Conclusion

In conclusion, CKRT is an alternative modality for dialysis in neonates with low body weight. The use of an appropriate catheter diameter and the establishment of vascular access *via* the internal jugular vein can enhance the circuit life of CKRT. An adequate anticoagulation method will increase circuit life span. The administration of relative hypertonic or colloid fluid infusions, and vasoactive agents, during the initial CKRT session can reduce the risk of intradialytic hypotension. The delicate removal of fluid is also important. Herein, we provide vital information relating to the delicate treatment of AKI in neonates with low body weight.

## Data Availability Statement

The original contributions presented in the study are included in the article/Supplementary Material, further inquiries can be directed to the corresponding author.

## Ethics Statement

The studies involving human participants were reviewed and approved by Institutional Review Board of National Cheng Kung University Hospital. Written informed consent from the participants' legal guardian/next of kin was not required to participate in this study in accordance with the national legislation and the institutional requirements. Written informed consent was obtained from the individual(s), and minor(s)' legal guardian/next of kin, for the publication of any potentially identifiable images or data included in this article.

## Author Contributions

C-YW was a resident on the pediatric team and drafted most of the case report, carried out the literature searches, and collected the data. Y-CL was an attending pediatric neonatologist and provided treatment and follow-up for the patients throughout the course of illness and gave suggestions and comments to polish this manuscript. C-CC was an attending pediatric nephrologist and edited/revised the manuscript. All authors approved the final manuscript as submitted and agree to be accountable for all aspects of the work.

## Conflict of Interest

The authors declare that the research was conducted in the absence of any commercial or financial relationships that could be construed as a potential conflict of interest.

## Publisher's Note

All claims expressed in this article are solely those of the authors and do not necessarily represent those of their affiliated organizations, or those of the publisher, the editors and the reviewers. Any product that may be evaluated in this article, or claim that may be made by its manufacturer, is not guaranteed or endorsed by the publisher.
