# Supplementary material for: A Case Series: Continuous Kidney Replacement Therapy in Neonates With Low Body Weight
Source: Front Pediatr. 2021 Nov 17;9:769220. doi: 10.3389/fped.2021.769220 (PMC8635746; doi:10.3389/fped.2021.769220)
Supplement: Supplementary file 1 [file Data_Sheet_1.docx]

Supplemental Material

Supplemental Table 1. Summary of the studies representing continuous kidney replacement therapy of infants.

**Supplemental Table 2.** Differences between dialysis machine used in our hospital and novel machines.

Supplemental References

**Supplemental Table 1. Summary of the studies representing continuous kidney replacement therapy of infants.**

| **Study** | **Modality** | **Body weights when CKRT** | **Vascular access site** | **Catheter** | **Bleeding complications** | **Hypotension incidences** | **Survival rates^*^** |
| --- | --- | --- | --- | --- | --- | --- | --- |
| Leyh 1996 (1) | CVVH | 3.2 to 5 kg | FV | 16G SL | None | NA | 7/12 |
| Picca 2001 (2) | CAVHD  CVVHD  HD | 2.4 to 4 kg | CAVHD: FA and FV  CVVHD: FV | CAVHD: 18G SL  CAVHD: 6.5-Fr DL | 1 pulmonary hemorrhage | 20%^**^ | 5/10 |
| Westrope 2010 (3) | CKRT | 1.7 to 3.7 kg | NA | 5-Fr and 6.5-Fr DL | None | 50% | 9/14 |
| [Askenazi](https://www.ncbi.nlm.nih.gov/pubmed/?term=Askenazi%20DJ%5BAuthor%5D&cauthor=true&cauthor_uid=23102589) 2013 (4) | CVVH  CVVHD  CVVHDF | 1.3 to 10 kg | FV, IJV, SCV | 5~12.5-Fr catheters | NA | NA | 34/84 |
| Mok 2017 (5) | CKRT | 1.9 to 3.6 kg | NA | NA | NA | 16 % | 5/12 |
| [Noh](https://www.ncbi.nlm.nih.gov/pubmed/?term=Noh%20ES%5BAuthor%5D&cauthor=true&cauthor_uid=31538434) 2019 (6) | CVVH | 1.2 to 8.4 kg | IJV | 6.5-Fr DL | 1 catheter-site bleeding | 33% | 5/33 |

Abbreviations: CAVHD: continuous arteriovenous hemodialysis, CKRT: continuous kidney replacement therapy, CVVH: continuous venovenous hemofiltration, CVVHD: continuous venovenous hemodialysis, DL: double lumen catheters, FA: femoral artery, FV: femoral vein, HD: hemodialysis, IJV: internal jugular vein, NA: not available, SCV: subclavian vein, SL: single lumen catheters.

^*^ The survival rates were represented as (numbers of patients who survived to discharge)/ (total participants). ^**^ Two patients among ten participants received HD and experienced hypotension.

**Supplemental Table 2. Differences between dialysis machine used in our hospital and novel machines**

| **Machine** | **FDA approval Year** | **Applicable population** | **Extracorporeal volume** | **Blood flow rate** |
| --- | --- | --- | --- | --- |
| Infomed HF400 (7) | 1999 | neonates to adults | 50-107 ml | 4-450 ml/min |
| CARPEDIEM (8; 9) | 2020 | infants weighted 2.5- 10 kg | 27.2-41.5 ml | 5-50 ml/min |
| Nidus (10; 11) | - | infants weighted 0.8- 8 kg | <10 ml | 20-45 ml/min |

**Supplemental References**

[1] Leyh R G, Nötzold A, Kraatz E G, Sievers H H, and Bernhard A. Continuous venovenous haemofiltration in neonates with renal insufficiency resulting from low cardiac output syndrome after cardiac surgery. *Cardiovasc Surg.* (1996) 4: 520-5. doi: 10.1016/0967-2109(95)00125-5

[2] Picca S, Dionisi-Vici C, Abeni D, Pastore A, Rizzo C, Orzalesi M, et al. Extracorporeal dialysis in neonatal hyperammonemia: modalities and prognostic indicators. *Pediatr Nephrol.* (2001) 16: 862-7. doi: 10.1007/s004670100702

[3] Westrope C, Morris K, Burford D, and Morrison G. Continuous hemofiltration in the control of neonatal hyperammonemia: a 10-year experience. *Pediatr Nephrol.* (2010) 25: 1725-30. doi: 10.1007/s00467-010-1549-3

[4] Askenazi D J, Goldstein S L, Koralkar R, Fortenberry J, Baum M, Hackbarth R, et al. Continuous renal replacement therapy for children ≤10 kg: a report from the prospective pediatric continuous renal replacement therapy registry. *J Pediatr.* (2013) 162: 587-592.e3. doi: 10.1016/j.jpeds.2012.08.044

[5] Diane Mok T Y, Tseng M H, Chiang M C, Lin J L, Chu S M, Hsu J F, et al. Renal replacement therapy in the neonatal intensive care unit. *Pediatr Neonatol.* (2018) 59: 474-480. doi: 10.1016/j.pedneo.2017.11.015

[6] Noh E S, Kim H H, Kim H S, Han Y S, Yang M, Ahn S Y, et al. Continuous Renal Replacement Therapy in Preterm Infants. *Yonsei Med J.* (2019) 60: 984-991. doi: 10.3349/ymj.2019.60.10.984

[7] J.F. Maher. (1989). “Machines for continuous renal replacement therapies,” in Replacement of Renal Function by Dialysis, ed. C. Ronco, R. Bellomo and A. Brendolan, (Springer, Dordrecht), 469-490.

[8] Ronco C, Garzotto F, Brendolan A, Zanella M, Bellettato M, Vedovato S, et al. Continuous renal replacement therapy in neonates and small infants: development and first-in-human use of a miniaturised machine (CARPEDIEM). *Lancet.* (2014) 383: 1807-13. doi: 10.1016/s0140-6736(14)60799-6

[9] Ronco C, Garzotto F, and Ricci Z. CA.R.PE.DI.E.M. (Cardio-Renal Pediatric Dialysis Emergency Machine): evolution of continuous renal replacement therapies in infants. A personal journey. *Pediatr Nephrol.* (2012) 27: 1203-11. doi: 10.1007/s00467-012-2179-8

[10] Coulthard M G, Crosier J, Griffiths C, Smith J, Drinnan M, Whitaker M, et al. Haemodialysing babies weighing <8 kg with the Newcastle infant dialysis and ultrafiltration system (Nidus): comparison with peritoneal and conventional haemodialysis. *Pediatr Nephrol.* (2014) 29: 1873-81. doi: 10.1007/s00467-014-2923-3

[11] B.A. Warady. (2012). “Maintenance Hemodialysis During Infancy,” in Pediatric Dialysis, ed. S. J. Swartz and F. Paglialonga, (Springer, Dordrecht), 379-387.
